# Supplementary material for: Reliability of Repeated Measures of Nutrient Intake by Diet Records in Residents in the Western Region of Japan
Source: Nutrients. 2019 Oct 18;11(10):2515. doi: 10.3390/nu11102515 (PMC6835429; doi:10.3390/nu11102515)
Supplement: Supplementary file 1 [file nutrients-11-02515-s001.pdf]

Table S1. Mean and SD of nutrient intakes in the current study and the Japan National Health and Nutrition Study 2016*

Nutrients	Current Study (n=131)		National Health and Nutrition Survey Japan 2016 (n=26,133)
	Mean [a]	SD	Mean
Energy kcal	**1846**	354	1865
Protein g	**69.9**	15.0	68.5
Lipid g	**56.5**	15.9	57.2
SFA[b] g	**15.65**	4.99	15.74
MUSFA[c] g	**20.51**	6.52	19.70
PUFA[d] g	**12.34**	3.31	NA
n-3PUFA g	**2.35**	0.87	2.16
n-6PUFA g	**9.94**	2.71	9.16
Cholesterol mg	**322**	107	311
Carbohydrate g	**247**	49	253
Total fiber g	**15.4**	4.8	14.2
Soluble fiber g	**3.4**	1.2	3.3
Insoluble fiber g	**11.3**	3.5	10.3
Alcohol[e] g	**7.5**	14.6	5.0*
Vitamin A µgRE[f]	**561**	353	524
Vitamin D µg	**7.9**	4.2	7.5
alpha-Tocopherol mg	**7.1**	1.9	6.4
Vitamin K µg	**232**	103	225
Vitamin B1mg	**0.94**	0.21	0.86
Vitamin B2 mg	**1.39**	0.35	1.15
Niacin mgNE[g]	**30.9**	7.2	14.4
Vitamin B6 mg	**1.34**	0.37	1.11
Vitamin B12 µg	**6.5**	3.3	6.0
Folate µg	**410**	128	277
Pantothenic acid mg	**6.08**	1.39	5.45
Vitamin C mg	**143**	55	89
Sodium mg	**3624**	856	3770
Salt-Eq g	**9.1**	2.2	9.6
Potassium mg	**2794**	732	2219
Calcium mg	**538**	164	502
Magnesium mg	**278**	67	238
Phosphorus mg	**1035**	232	976
Iron mg	**8.7**	2.3	7.4
Selenium µg	**62**	18	NA
Zinc mg	**7.9**	1.7	8.0
Cupper mg	**1.21**	0.30	1.11
Manganese mg	**4.35**	1.29	NA
beta-carotene µg	**3469**	1696	NA
alpha-carotene µg	**563**	350	NA
beta-cryptoxanthin µg	**528.4**	442.7	NA
% energy intake from carbohydrate	**53.5**		54.2
% energy intake from protein	**15.2**		14.7
% energy intake from lipid	**27.5**		27.6

*The data in this study was collected between 2014 and 2015 in Nagasaki Prefecture, Japan. The title of this study is "Reliability of repeated measures of nutrient intake by diet records in residents in the western region of Japan."
[a]Means of 1571 diet records: an average of 12 records per participant
[b]Saturated fatty acid
[c]Monounsaturated fatty acid
[d]Polyunsaturated fatty acid
[e]The total alcohol intake in ethanol in gram was estimated from the total alcohol (ethanol) intake of beverages (beer, saki wine, and liquor and others) reported in the NHNS 2016.
[f]Retinol equivalent
[g]Niacin equivalent

Table S2. Mean and SD of nutrient intakes by the survey order from the 12 diet recalls*

Nutrients	Day 1		Day2		Day 3		Day 4		Day 5		Day 6		Day 7		Day 8		Day 9		Day 10		Day 11		Day 12	
	n=131		n=131		n=131		n=131		n=131		n=131		n=131		n=131		n=131		n=131		n=131		n=130 [a]	
	Mean	SD	Mean	SD	Mean	SD	Mean	SD	Mean	SD	Mean	SD	Mean	SD	Mean	SD	Mean	SD	Mean	SD	Mean	SD	Mean	SD
Energy kcal	**1872**	498	**1843**	451	**1844**	464	**1840**	449	**1875**	464	**1817**	452	**1854**	498	**1855**	496	**1861**	503	**1853**	538	**1806**	541	**1833**	480
Protein g	**71.5**	21.6	**70.2**	20.0	**69.4**	19.5	**72.4**	21.5	**70.00**	20.50	**68.62**	19.93	**69.7**	19.0	**67.9**	21.5	**69.7**	22.5	**70.3**	22.9	**70.1**	24.0	**69.4**	21.3
Lipid g	**57.6**	23.2	**57.2**	24.2	**55.9**	25.8	**57.6**	25.8	**59.05**	24.45	**54.75**	22.37	**57.0**	25.0	**56.6**	23.3	**58.1**	26.8	**55.4**	25.2	**53.6**	24.4	**55.2**	23.0
SFA[b] g	**15.70**	7.18	**15.88**	8.16	**15.27**	8.27	**15.58**	7.97	**16.13**	7.30	**15.24**	7.27	**16.36**	8.37	**15.61**	8.19	**16.25**	8.98	**15.24**	7.88	**14.67**	7.41	**15.90**	8.00
MSFA[c] g	**20.96**	9.92	**21.05**	11.11	**20.01**	10.66	**20.84**	10.93	**21.74**	10.61	**20.09**	9.54	**20.70**	10.19	**20.20**	9.31	**21.17**	11.53	**20.31**	11.00	**19.44**	10.59	**19.65**	8.93
PUFA[d] g	**13.02**	5.68	**12.30**	4.71	**12.80**	6.43	**13.17**	6.92	**13.16**	6.39	**11.51**	5.12	**11.91**	5.38	**12.71**	5.56	**12.42**	5.39	**12.19**	5.59	**11.43**	4.93	**11.45**	4.98
n-6PUFA g	**2.25**	1.21	**2.33**	1.58	**2.46**	1.59	**2.43**	1.61	**2.29**	1.44	**2.03**	1.37	**2.22**	1.62	**2.64**	1.80	**2.56**	1.67	**2.40**	1.76	**2.30**	1.69	**2.29**	1.77
n-3PUFA g	**10.71**	4.97	**9.93**	3.86	**10.30**	5.52	**10.69**	5.82	**10.84**	5.58	**9.43**	4.28	**9.65**	4.60	**10.02**	4.66	**9.81**	4.57	**9.75**	4.55	**9.08**	4.05	**9.11**	4.17
Cholesterol mg	**316**	180	**328**	177	**315**	177	**336**	184	**338**	190	**334**	185	**313**	175	**294**	172	**327**	187	**324**	182	**322**	199	**321**	184
Carbohydrates g	**248**	72	**244**	65	**249**	65	**242**	61	**247**	59	**246**	65	**248**	69	**249**	71	**251**	71	**250**	73	**244**	77	**246**	70
Total fiber g	**16.7**	7.3	**16.4**	6.8	**14.7**	5.8	**14.8**	5.9	**15.5**	5.8	**14.8**	5.5	**14.1**	5.4	**14.9**	6.1	**15.4**	7.0	**16.4**	8.6	**15.5**	6.3	**15.4**	6.6
Soluble fiber g	**3.7**	1.7	**3.5**	1.8	**3.1**	1.4	**3.2**	1.5	**3.4**	1.5	**3.3**	1.3	**3.1**	1.3	**3.4**	2.3	**3.3**	1.5	**3.8**	3.1	**3.5**	1.6	**3.4**	1.7
Insoluble fiber g	**12.0**	5.2	**11.8**	4.9	**10.8**	4.6	**10.9**	4.3	**11.3**	4.3	**10.7**	4.2	**10.4**	4.0	**10.9**	4.4	**11.6**	5.5	**12.0**	5.7	**11.4**	4.9	**11.3**	5.0
Alcohol g	**8.2**	20.5	**7.7**	20.3	**6.9**	14.8	**6.6**	16.1	**8.1**	18.9	**6.8**	15.0	**7.5**	18.0	**8.7**	24.8	**5.9**	14.5	**8.4**	19.5	**7.0**	15.7	**8.2**	20.2
Vitamin A µgRE[e]	**681**	1431	**572**	572	**491**	425	**721**	1925	**549**	807	**566**	962	**434**	288	**412**	229	**673**	1390	**498**	275	**626**	1265	**510**	308
Vitamin D µg	**6.1**	6.8	**7.5**	9.1	**7.5**	9.1	**8.2**	11.4	**7.3**	8.7	**7.5**	8.1	**9.1**	12.1	**9.4**	10.2	**8.7**	9.1	**7.0**	7.4	**7.5**	9.2	**8.3**	11.2
alpha-tocopherol mg	**7.1**	2.9	**7.5**	3.1	**7.1**	3.1	**6.8**	3.1	**7.7**	3.7	**7.3**	3.0	**7.0**	3.1	**6.8**	2.8	**7.4**	3.4	**6.8**	3.0	**6.8**	3.1	**6.9**	3.1
Vitamin K µg	**279**	177	**281**	204	**204**	118	**214**	137	**228**	164	**201**	164	**198**	149	**202**	156	**223**	150	**242**	153	**243**	157	**266**	183
Vitamin B_1 mg	**0.95**	0.35	**0.94**	0.37	**0.87**	0.37	**0.95**	0.41	**0.98**	0.38	**0.89**	0.33	**0.91**	0.34	**0.90**	0.41	**0.94**	0.44	**0.96**	0.40	**0.98**	0.45	**0.96**	0.41
Vitamin B_2 mg	**1.40**	0.49	**1.40**	0.47	**1.35**	0.41	**1.44**	0.53	**1.37**	0.45	**1.34**	0.46	**1.33**	0.48	**1.34**	0.51	**1.42**	0.54	**1.38**	0.52	**1.43**	0.62	**1.44**	0.53
Niacin mgNE[f]	**31.8**	10.3	**30.2**	8.9	**30.8**	10.4	**32.1**	10.5	**30.74**	10.00	**30.25**	10.25	**30.7**	9.5	**29.9**	10.4	**31.7**	12.0	**31.4**	12.0	**30.9**	11.0	**30.7**	10.3
Vitamin B_6 mg	**1.36**	0.46	**1.31**	0.46	**1.29**	0.43	**1.38**	0.51	**1.35**	0.52	**1.32**	0.49	**1.34**	0.51	**1.29**	0.51	**1.42**	0.58	**1.38**	0.58	**1.34**	0.52	**1.31**	0.47
Vitamin B_{12} µg	**6.0**	8.0	**6.2**	6.9	**6.1**	5.8	**7.4**	9.0	**5.03**	4.79	**5.95**	5.27	**5.6**	5.6	**7.0**	7.3	**8.4**	9.1	**7.2**	7.8	**6.4**	7.6	**6.6**	7.4
Folate µg	**471**	234	**452**	187	**389**	148	**425**	240	**389**	159	**376**	169	**360**	136	**367**	149	**409**	210	**415**	171	**433**	194	**430**	180
Pantothenic acid mg	**6.16**	2.19	**5.89**	1.59	**5.89**	1.59	**6.21**	2.34	**6.16**	1.81	**6.10**	1.87	**5.99**	1.83	**5.76**	1.89	**6.30**	2.19	**6.19**	1.84	**6.19**	2.19	**6.11**	2.08
Vitamin C mg	**155**	80	**160**	86	**132**	61	**138**	69	**124**	67	**130**	67	**124**	63	**126**	73	**158**	90	**158**	87	**160**	84	**148**	79
Sodium mg	**3833**	1403	**3596**	1343	**3489**	1192	**3430**	1336	**3625**	1342	**3690**	1338	**3732**	1349	**3663**	1262	**3483**	1423	**3649**	1489	**3558**	1439	**3736**	1428
Salt-Eq g	**9.6**	3.6	**9.1**	3.4	**8.8**	3.1	**8.6**	3.4	**9.14**	3.42	**9.30**	3.42	**9.4**	3.4	**9.2**	3.2	**8.8**	3.6	**9.2**	3.8	**8.9**	3.7	**9.4**	3.6
Potassium mg	**2981**	1064	**2872**	943	**2717**	866	**2800**	897	**2792**	882	**2717**	873	**2665**	826	**2621**	811	**2837**	1033	**2919**	1125	**2796**	980	**2811**	1014
Calcium mg	**578**	277	**546**	254	**517**	229	**546**	234	**528**	226	**497**	218	**521**	217	**542**	269	**557**	257	**546**	265	**526**	241	**549**	250
Magnesium mg	**311**	135	**299**	141	**266**	85	**277**	89	**276**	85	**268**	81	**270**	79	**264**	82	**275**	99	**275**	97	**273**	93	**282**	120
Phosphorus mg	**1059**	321	**1030**	303	**1013**	290	**1086**	331	**1062**	318	**1022**	285	**1035**	294	**1007**	318	**1043**	344	**1026**	334	**1015**	347	**1021**	313
Iron mg	**9.6**	4.2	**8.9**	2.9	**8.6**	2.9	**9.0**	3.2	**8.45**	2.89	**8.34**	2.99	**7.9**	2.7	**8.2**	3.0	**9.1**	3.5	**8.9**	3.3	**8.9**	3.8	**8.6**	3.3
Selenium µg	**57**	29	**66**	37	**63**	38	**59**	30	**61**	35	**61**	29	**60**	33	**57**	31	**65**	41	**64**	40	**62**	38	**64**	37
Zinc mg	**8.3**	2.8	**8.2**	2.9	**7.7**	2.3	**7.9**	2.6	**7.94**	2.29	**7.68**	2.07	**8.0**	2.4	**7.3**	2.2	**8.0**	3.0	**8.2**	3.4	**8.3**	3.5	**7.8**	2.4
Cupper mg	**1.25**	0.51	**1.21**	0.39	**1.18**	0.36	**1.22**	0.39	**1.20**	0.37	**1.21**	0.42	**1.21**	0.37	**1.19**	0.39	**1.23**	0.44	**1.23**	0.43	**1.22**	0.58	**1.19**	0.39
Manganese mg	**4.57**	1.69	**4.50**	1.75	**4.40**	1.67	**4.46**	1.66	**4.04**	1.51	**3.96**	1.60	**4.13**	1.68	**4.29**	1.82	**4.43**	1.85	**4.44**	1.65	**4.54**	1.66	**4.46**	1.73
beta-carotene µg	**4348**	4356	**4072**	3447	**3236**	2341	**2982**	2341	**3610**	2945	**3573**	2613	**2912**	2159	**2895**	2295	**3508**	3051	**3484**	2655	**3384**	2599	**3598**	2966
alpha-carotene µg	**804**	1126	**588**	651	**605**	636	**494**	580	**504**	569	**566**	727	**456**	530	**474**	581	**466**	560	**573**	562	**601**	660	**629**	685
beta-cryptoxanthin µg	**682.3**	1123.7	**551.9**	927.5	**465.5**	876.6	**251.3**	606.8	**203.76**	418.85	**108.23**	333.43	**64.0**	174.8	**222.3**	494.8	**843.3**	1145.5	**1044.4**	1310.3	**992.5**	1255.3	**906.1**	1277.4

*The data in this study was collected between 2014 and 2015 in Nagasaki Prefecture, Japan. The title of this study is "Reliability of repeated measures of nutrient intake by diet records in residents in the western region of Japan."

[a]One participant completed 11 diet records.
[b]Saturated fatty acid
[c]Monounsaturated fatty acid
[d]Polyunsaturated fatty acid
[e]Retinol equivalent
[f]Niacin equivalent

Table S3. Sex-specific Intraclass Correlation Coefficients (ICC) and the ratio of within- to between person variance (σ_w^2/σ_b^2) for intakes of energy-adjusted nutrients*[a]

Nutrients	Male (n=33)				Female (n=98)			
	Mean[b]	SD	ICC (95%CI)	$R_{w/b}$	Mean[c]	SD	ICC (95%CI)	$R_{w/b}$
Energy kcal	2091	369	0.40 (0.28, 0.54)	1.5	1764	310	0.46 (0.38, 0.54)	1.2
Protein g	77.5	16.3	0.32 (0.20, 0.46)	2.1	67.4	13.7	0.28 (0.22, 0.36)	2.6
Lipid g	62.3	15.9	0.37 (0.25, 0.51)	1.7	54.6	15.6	0.25 (0.19, 0.33)	3.0
SFA[d] g	16.85	4.91	0.35 (0.23, 0.49)	1.9	15.25	4.97	0.27 (0.21, 0.35)	2.7
MSFA[e] g	23.03	6.78	0.32 (0.20, 0.45)	2.1	19.67	6.24	0.25 (0.19, 0.32)	3.0
PUFA[f] g	13.82	3.21	0.15 (0.08, 0.28)	5.7	11.84	3.20	0.14 (0.09, 0.20)	6.1
n-6PUFA g	2.78	0.93	0.15 (0.08, 0.27)	5.7	2.21	0.80	0.13 (0.09, 0.19)	6.7
n-3PUFA g	10.99	2.66	0.09 (0.04, 0.21)	10.1	9.59	2.64	0.14 (0.10, 0.20)	6.1
Cholesterol mg	375	127	0.22 (0.13, 0.35)	3.5	305	93	0.10 (0.06, 0.16)	9.0
Carbohydrates g	261	65	0.47 (0.34, 0.61)	1.1	242	42	0.29 (0.22, 0.37)	2.4
Total fiber g	14.2	5.7	0.54 (0.40, 0.67)	0.9	15.8	4.4	0.42 (0.34, 0.50)	1.4
Soluble fiber g	3.0	1.3	0.48 (0.35, 0.61)	1.1	3.5	1.1	0.25 (0.19, 0.32)	3.0
Insoluble fiber g	10.5	4.4	0.54 (0.41, 0.67)	0.9	11.5	3.2	0.40 (0.33, 0.48)	1.5
vitamin A µgRE[g]	547	409	0.07 (0.03, 0.18)	13.3	566	335	0.01 (0.00, 0.12)	99.0
Vitamin D µg	9.6	5.3	0.11 (0.05, 0.22)	8.1	7.3	3.6	0.03 (0.01, 0.08)	32.3
alpha-Tocopherol mg	7.1	2.0	0.25 (0.15, 0.38)	3.0	7.1	1.8	0.21 (0.15, 0.28)	3.8
Vitamin K µg	230	106	0.29 (0.18, 0.43)	2.4	232	103	0.33 (0.26, 0.41)	2.0
Vitamin B_1 mg	0.98	0.22	0.11 (0.05, 0.22)	8.1	0.92	0.2	0.12 (0.08, 0.18)	7.3
Vitamin B_2 mg	1.42	0.36	0.24 (0.14, 0.38)	3.2	1.37	0.35	0.32 (0.25, 0.40)	2.1
Niacin mgNE[h]	35.8	8.2	0.29 (0.18, 0.43)	2.4	29.3	6.1	0.19 (0.14, 0.26)	4.3
Vitamin B_6 mg	1.44	0.44	0.46 (0.33, 0.60)	1.2	1.31	0.34	0.37 (0.30, 0.45)	1.7
Vitamin B_{12} µg	7.4	4.2	0.16 (0.08, 0.28)	5.3	6.2	2.9	0.05 (0.02, 0.10)	19.0
Folate µg	399	154	0.41 (0.28, 0.55)	1.4	413	118	0.30 (0.24, 0.38)	2.3
Pantothenic acid mg	6.34	1.59	0.37 (0.25, 0.52)	1.7	5.99	1.32	0.29 (0.22, 0.36)	2.4
Vitamin C mg	126	58	0.40 (0.27, 0.54)	1.5	149	53	0.35 (0.28, 0.43)	1.9
Sodium mg	3880	940	0.23 (0.14, 0.36)	3.3	3538	812	0.22 (0.16, 0.29)	3.5
Salt-Eq g	9.8	2.4	0.22 (0.13, 0.35)	3.5	8.9	2.1	0.22 (0.16, 0.29)	3.5
Potassium mg	2770	811	0.51 (0.38, 0.64)	1.0	2803	707	0.48 (0.41, 0.56)	1.1
Calcium mg	487	170	0.36 (0.24, 0.50)	1.8	555	159	0.28 (0.21, 0.36)	2.6
Magnesium mg	286	69	0.20 (0.11, 0.33)	4.0	275	66	0.29 (0.22, 0.37)	2.4
Phosphorus mg	1100	252	0.40 (0.27, 0.54)	1.5	1013	222	0.35 (0.27, 0.43)	1.9
Iron mg	8.8	2.7	0.44 (0.31, 0.58)	1.3	8.7	2.2	0.31 (0.24, 0.39)	2.2
Selenium µg	69	19	0.06 (0.02, 0.17)	15.7	59	17	0.07 (0.04, 0.13)	13.3
Zinc mg	8.7	1.9	0.20 (0.11, 0.33)	4.0	7.7	1.5	0.16 (0.11, 0.23)	5.3
Cupper mg	1.27	0.37	0.19 (0.11, 0.32)	4.3	1.19	0.27	0.33 (0.26, 0.41)	2.0
Manganese mg	4.32	1.48	0.42 (0.30, 0.56)	1.4	4.36	1.22	0.45 (0.37, 0.53)	1.2
beta-Carotene µg	3362	1995	0.23 (0.14, 0.37)	3.3	3504	1594	0.24 (0.18, 0.32)	3.2
alpha-Carotene µg	593	461	0.06 (0.02, 0.17)	15.7	554	305	0.02 (0.00, 0.09)	49.0
beta-Cryptoxanthin µg	319	366	0.15 (0.08, 0.27)	5.7	599	446	0.03 (0.01, 0.08)	32.3

*The data in this study was collected between 2014 and 2015 in Nagasaki Prefecture, Japan. The title of this study is “Reliability of repeated measures of nutrient intake by diet records in residents in the western region of Japan.”

[a]Adjustment by use of the residual model method

[b]Mean of 395 diet records obtained from 33 male participants

[c]Means of 1176 diet records obtained from 98 female participants

[d]Saturated fatty acid

[e]Monounsaturated fatty acid

[f]Polyunsaturated fatty acid

[g]Retinol equivalent

[h]Niacin equivalent

Table S4. Coefficients of within-person variation and the number of days needed to lie within specified 10% of the true mean of energy-adjusted nutrients*[a]

Nutrients	Male (n=33)		Female (n=98)	
	CV_W (95% CI)[b]	Specified limit as % 10%	CV_W (95% CI)[c]	Specified limit as % 10%
Protein g	0.18 (0.17, 0.20)	13	0.17 (0.16, 0.18)	12
Lipid g	0.24 (0.22, 0.27)	23	0.23 (0.22, 0.24)	21
SFA[d] g	0.33 (0.29, 0.37)	42	0.31 (0.29, 0.33)	37
MSFA[e] g	0.32 (0.29, 0.36)	40	0.30 (0.29, 0.32)	35
PUFA[f] g	0.28 (0.26, 0.31)	31	0.32 (0.30, 0.34)	40
n-6PUFA g	0.33 (0.30, 0.36)	42	0.34 (0.33, 0.36)	45
n-3PUFA g	0.57 (0.51, 0.63)	125	0.55 (0.51, 0.58)	117
Cholesterol mg	0.45 (0.40, 0.51)	78	0.49 (0.46, 0.52)	93
Carbohydrates g	0.15 (0.14, 0.16)	9	0.12 (0.12, 0.13)	6
Total fiber g	0.30 (0.26, 0.35)	35	0.26 (0.25, 0.28)	26
Soluble fiber g	0.36 (0.32, 0.42)	50	0.41 (0.38, 0.44)	65
Insoluble fiber g	0.31 (0.27, 0.36)	37	0.27 (0.25, 0.29)	29
vitamin A µgRE[g]	1.32 (1.09, 1.61)	670	1.67 (1.49, 1.86)	1072
Vitamin D µg	1.11 (0.92, 1.34)	474	1.41 (1.27, 1.56)	764
alpha-Tocopherol mg	0.33 (0.30, 0.36)	42	0.32 (0.31, 0.34)	40
Vitamin K µg	0.62 (0.52, 0.73)	148	0.56 (0.51, 0.62)	121
Vitamin B_1 mg	0.38 (0.34, 0.41)	56	0.31 (0.30, 0.33)	37
Vitamin B_2 mg	0.26 (0.24, 0.29)	26	0.25 (0.23, 0.26)	25
Niacin mgNE[h]	0.23 (0.21, 0.25)	21	0.23 (0.21, 0.24)	21
Vitamin B_6 mg	0.24 (0.22, 0.27)	23	0.24 (0.23, 0.26)	23
Vitamin B_{12} µg	0.89 (0.75, 1.06)	305	1.33 (1.20, 1.48)	680
Folate µg	0.35 (0.31, 0.39)	48	0.34 (0.32, 0.36	45
Pantothenic acid mg	0.20 (0.19, 0.22)	16	0.21 (0.20, 0.22)	17
Vitamin C mg	0.46 (0.39, 0.54)	82	0.39 (0.37, 0.43)	59
Sodium mg	0.31 (0.28, 0.34)	37	0.28 (0.27, 0.30)	31
Salt-Eq g	0.32 (0.29, 0.35)	40	0.29 (0.27, 0.30)	33
Potassium mg	0.21 (0.19, 0.23)	17	0.19 (0.18, 0.21)	14
Calcium mg	0.36 (0.32, 0.41)	50	0.32 (0.31, 0.35)	40
Magnesium mg	0.30 (0.27, 0.33)	35	0.25 (0.24, 0.27)	25
Phosphorus mg	0.18 (0.16, 0.20)	13	0.17 (0.16, 0.18)	12
Iron mg	0.23 (0.21, 0.26)	21	0.26 (0.25, 0.28)	26
Selenium µg	0.50 (0.45, 0.55)	97	0.50 (0.47, 0.53)	97
Zinc mg	0.22 (0.20, 0.24)	19	0.23 (0.22, 0.24)	21
Cupper mg	0.37 (0.34, 0.42)	53	0.21 (0.20, 0.22)	17
Manganese mg	0.28 (0.25, 0.31)	31	0.26 (0.24, 0.28)	26
beta-carotene µg	0.85 (0.70, 1.03)	278	0.68 (0.61, 0.74)	178

alpha-carotene μg	1.99 (1.51, 2.61)	1522	2.57 (2.18, 3.03)	2538
beta-cryptoxanthin μg	2.10 (1.45, 3.06)	1695	2.37 (2.00, 2.79)	2158

*The data in this study was collected between 2014 and 2015 in Nagasaki Prefecture, Japan. The title of this study is "Reliability of repeated measures of nutrient intake by diet records in residents in the western region of Japan."

[a]Adjustment by use of the residual model method

[b]Analysis was done using 395 diet records obtained from 33 male participants.

[c]Analysis was done using 1176 diet records obtained from 98 female participants.

[d]Saturated fatty acid

[e]Monounsaturated fatty acid

[f]Polyunsaturated fatty acid

[g]Retinol equivalent

[h]Niacin equivalent
